# Functional investigation of two simultaneous or separately segregating *DSP* variants within a single family supports the theory of a dose‐dependent disease severity

**DOI:** 10.1111/exd.14571

**Published:** 2022-04-01

**Authors:** Mathilde C. S. C. Vermeer, Daniela Andrei, Duco Kramer, Albertine M. Nijenhuis, Yvonne M. Hoedemaekers, Helga Westers, Jan D. H. Jongbloed, Hendri H. Pas, Maarten P. van den Berg, Herman H. W. Silljé, Peter van der Meer, Maria C. Bolling

**Affiliations:** ^1^ Department of Cardiology University Medical Center Groningen University of Groningen Groningen The Netherlands; ^2^ Department of Dermatology University Medical Center Groningen University of Groningen Groningen The Netherlands; ^3^ Department of Genetics Radboud University Medical Center Radboud University Nijmegen Nijmegen The Netherlands; ^4^ Department of Genetics University Medical Center Groningen University of Groningen Groningen The Netherlands

**Keywords:** cardiocutaneous syndrome, cardiomyopathy and genotype–phenotype correlation, desmoplakin, palmoplantar keratoderma

## Abstract

Desmoplakin (DP*)* is an important component of desmosomes, essential in cell–cell connecting structures in stress‐bearing tissues. Over the years, many hundreds of pathogenic variants in *DSP* have been associated with different cutaneous and cardiac phenotypes or a combination, known as a cardiocutaneous syndrome. Of less than 5% of the reported *DSP* variants, the effect on the protein has been investigated. Here, we describe and have performed RNA, protein and tissue analysis in a large family where *DSP* ^c.273+5G>A/c.6687delA^ segregated with palmoplantar keratoderma (PPK), woolly hair and lethal cardiomyopathy, while *DSP* ^WT/c.6687delA^ segregated with PPK and milder cardiomyopathy. hiPSC‐derived cardiomyocytes and primary keratinocytes from carriers were obtained for analysis. Unlike the previously reported nonsense variants in the last exon of *DSP* that bypassed the nonsense‐mediated mRNA surveillance system leading to protein truncation, variant c.6687delA was shown to cause the loss of protein expression. Patients carrying both variants and having a considerably more severe phenotype were shown to have 70% DP protein reduction, while patients carrying only c.6687delA had 50% protein reduction and a milder phenotype. The analysis of RNA from patient cells did not show any splicing effect of the c.273+5G>A variant. However, a minigene splicing assay clearly showed alternative spliced transcripts originating from this variant. This study shows the importance of RNA and protein analyses to pinpoint the exact effect of *DSP* variants instead of solely relying on predictions. In addition, the particular pattern of inheritance, with simultaneous or separately segregating *DSP* variants within the same family, strongly supports the theory of a dose‐dependent disease severity.

## 1 BACKGROUND

Desmosomes maintain cell–cell cohesion by forming dense intercellular bonds that anchor the cell's intermediate filaments to the cell membrane in skin and heart.^1^ Pathogenic variants in its major constituent desmoplakin (DP; gene *DSP*) may give isolated cardiomyopathy, skin fragility, palmoplantar keratoderma (PPK) or woolly hair (WH), but also a combination of these, like in Carvajal syndrome.^2^, ^3^ In skin, *DSP* produces nearly equivalent levels of two isoforms: DPI and the shorter DPII isoform, while the heart predominantly contains DPI.^4^, ^5^ In 2000, homozygous variants causing c‐terminally truncated DP were shown to cause Carvajal syndrome,^6^ but also dominant loss‐of‐function variants have been linked to a combination of mild PPK, WH and cardiomyopathy.^7^ Over the years, hundreds of *DSP* variants have been associated with disease, yet only in a very few studies, the effect of these variants on RNA, protein and the cells/tissues involved has been investigated, leaving the genotype–phenotype correlation poorly understood.^7^, ^8^ Most studies merely rely on the use of *in silico* models to predict variant‐outcome. Analysis of hiPSC‐derived cardiomyocytes as well as primary keratinocytes is hardly performed, but could be the essential stepping stone to rightfully denote the effects of *DSP* variants and a better understanding of the genotype–phenotype correlation.

## 2 QUESTIONS ADDRESSED

We describe a large family where members show various expression of moderate to lethal cardiomyopathy, PPK and WH. In this family, two variants in *DSP* (c.273+5G>A and c.6687delA) were identified. Previously, we investigated engineered heart tissues, derived from the most severely affected proband carrying both variants in trans.^9^ Here, we investigate segregation of these *DSP* variants among other affected relatives. We functionally assessed the consequences of these variants on RNA and protein levels in cardiomyocytes and primary keratinocytes from several affected family members and compared these data to *in silico* predictions, the various heart and skin disease severities and the sparse amount of experimental studies on other *DSP* variants.

## 3 EXPERIMENTAL DESIGN

Patients' written informed consent was received prior to this study. A gene panel for inherited cardiomyopathies was performed on the proband, and segregation of variants was confirmed in other affected family members. The effects of variants were predicted using *in silico* prediction software. A minigene splicing assay was deployed and skin biopsies from non‐lesional, upper arm skin were obtained from *DSP* ^c.273+5G>A/c.6687delA^ and *DSP* ^WT/c.6687delA^ carriers. Unfortunately, the *DSP* ^c.273+5G>A/WT^ carrier was not willing to participate in the study. Biopsy‐derived keratinocytes were cultured on flexible stretch plates with and without uniaxial stretching regimes. Immunofluorescence microscopy and electron microscopy were performed on skin biopsies and cultured keratinocytes, in addition to cardiomyocytes as previously described.^9^ RNA and protein were extracted and quantitatively assessed and compared with controls. An extensive Material and Methods section can be found in the online supplements.

## 4 RESULTS

### 4.1 Segregation of *DSP* variants in affected family members

**FIGURE 1:**
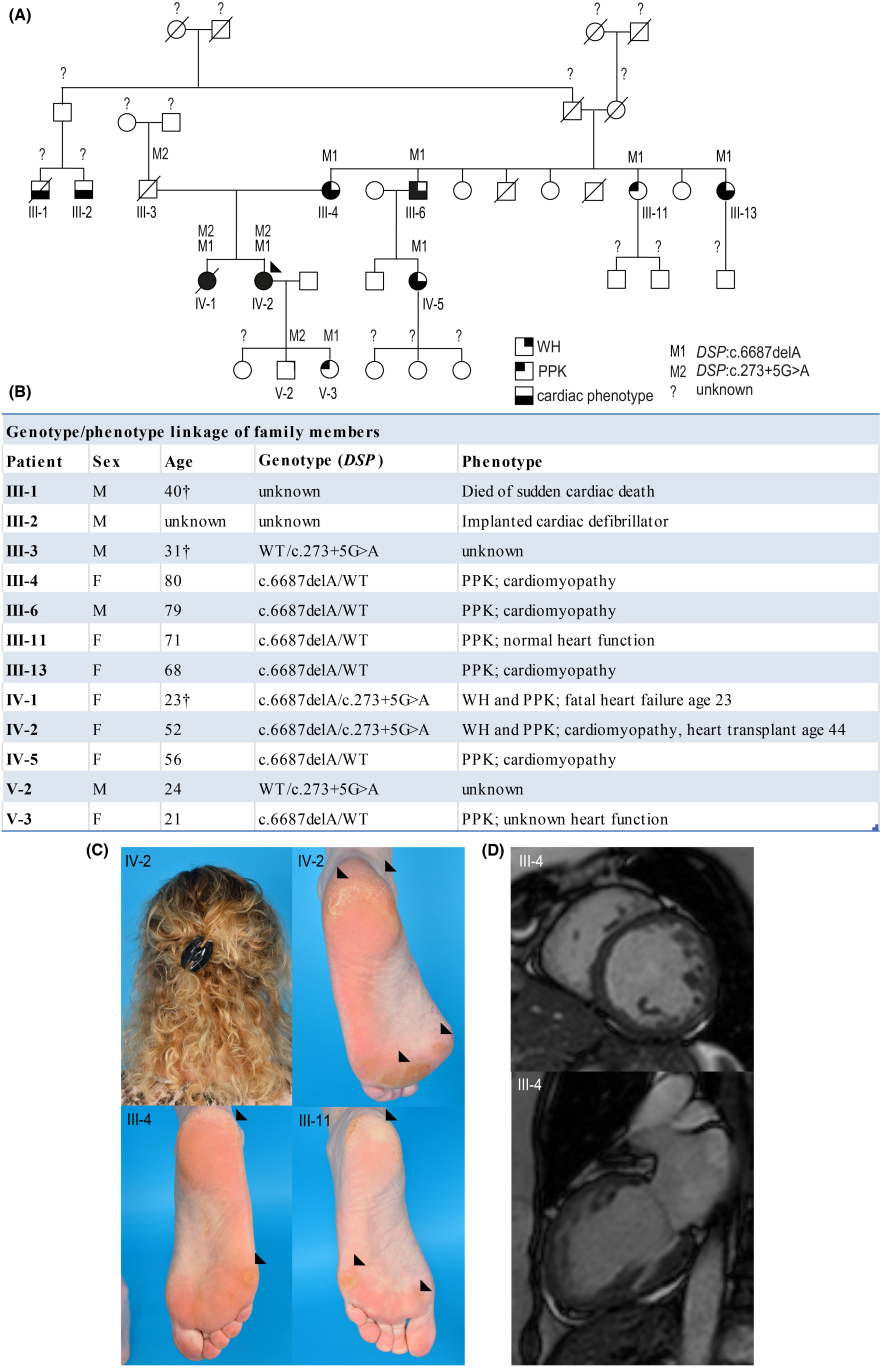
Clinical assessment of a Dutch family with segregating *DSP* variants. (A) Pedigree of a Dutch family with segregating *DSP* variants (M1 and M2). PPK = palmoplantar keratoderma; WH = woolly hair; “?” = unknown genotype/phenotype; the arrow indicates index patient IV‐2. (B) Table of information for affected family members. (C) Index patient IV‐2 shown left with mild WH and right with feet showing focal PPK. Focal PPK is additionally shown in patient III‐4 and III‐11. The arrows point towards the formation of excessive callus at pressure points. (D) Cardiac MRI of patient III‐4, showing dilation of and trabeculations in the left ventricle. *Of note: patients III:4, III‐11, IV‐2 and V‐3 have provided the biopsies used in this study*

A 52‐year‐old woman who underwent heart transplantation at the age of 44 because of a progressive and life‐threatening cardiomyopathy was referred to a dermatologist. WH and a mild focal PPK without skin fragility (Figure 1A; IV‐2) were observed. Her similarly affected sister (IV‐1) died from sudden heart failure at the age of 23 years, while several 3rd and 4th generation family members on the maternal side were diagnosed with cardiomyopathy and PPK. Next‐generation sequencing with a targeted cardiac gene panel had revealed two *DSP* variants in the index patient: c.273+5G>A^10^ and c.6687delA.^9^, ^11^ Skin biopsies were obtained after ethical approval and informed consent. The paternally derived splice site variant, c.273+5G>A, was detected in four family members, whereas the maternally derived c.6687delA variant was found in eight (Figure 1A‐B). Overall, compound heterozygosity of *DSP* ^c.273+5G>A/c.6687delA^ segregated in 2/2 carriers with mild WH, PPK and early onset, severe cardiomyopathy, whereas heterozygosity of *DSP* ^WT/c.6687delA^ segregated in 6/6 carriers with PPK and in 4/6 carriers with cardiomyopathy (Figure 1B–D). The only living *DSP* ^c.273+5G>A/WT^ carrier was unwilling to participate, while the other carrier died from an accident at age 31. Whether *DSP* ^c.273+5G>A/WT^ segregates with a phenotype in this family thus remains unknown. Family members without any variant did not reveal any of the aforementioned phenotypic features. Screening of variant c.273+5G>A in whole‐exome sequencing data of a cardiomyopathy cohort identified 11 additional carriers from 8 other families (Table S1).

### 4.2 Functional assessment of *DSP* variants in patient keratinocytes and cardiomyocytes

**FIGURE 2:**
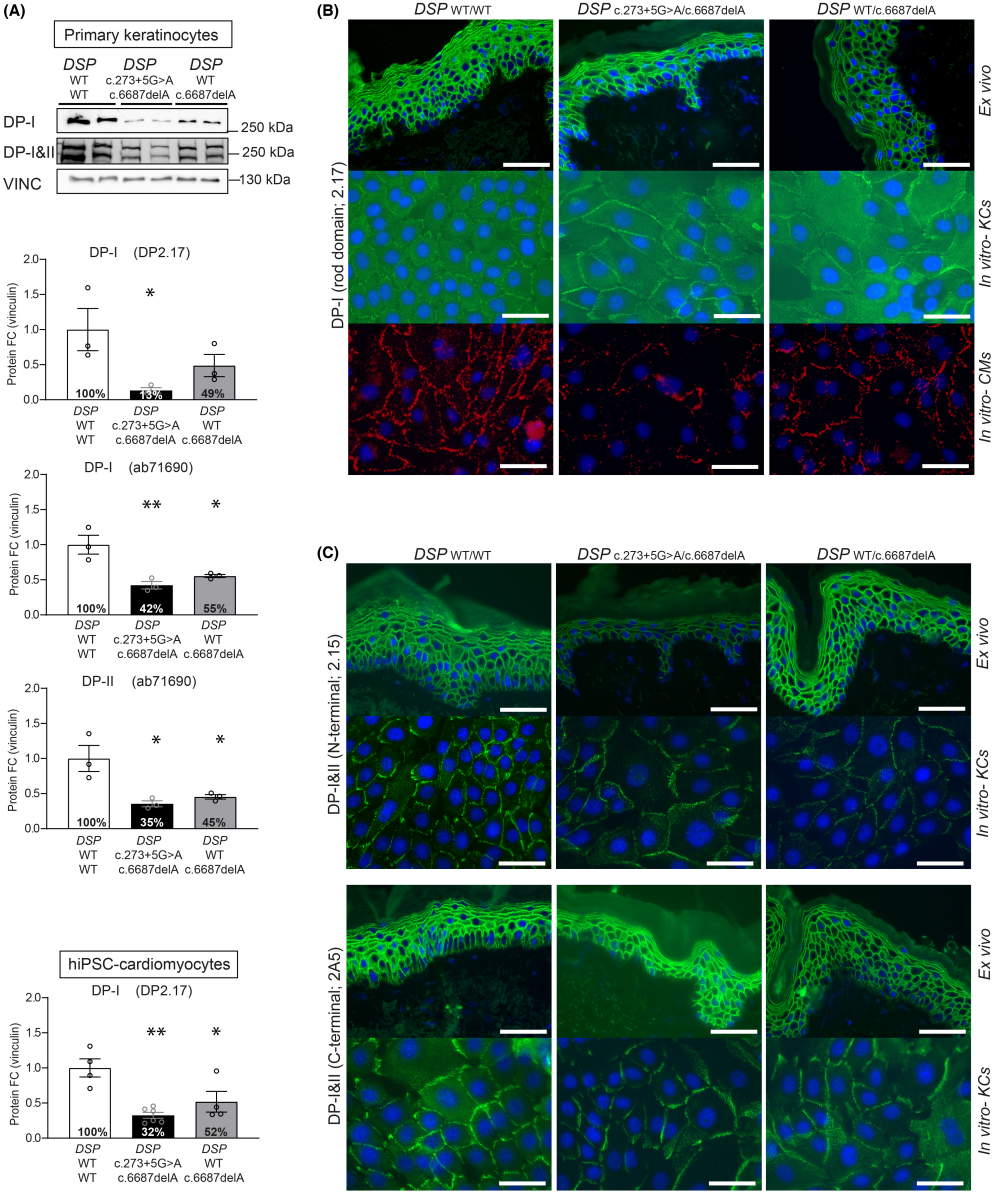
DP expression in non‐palmoplantar *ex vivo* skin and *in vitro* keratinocytes and hiPSC‐derived cardiomyocytes. (A) Western blots of desmoplakin protein expression using different antigen maps to DP‐I and DP‐I&II in compound heterozygous *(DSP* ^c.273+5G>A/c.6687delA^) and heterozygous (*DSP* ^WT/c.6687delA^) carrier in comparison with control. Quantified protein levels of DP isoform‐I and II, using different antibodies in cultured primary keratinocytes. 2 controls (*n* = 3), 1 patient of each genotype (*n* = 3/patient). Also, quantified the protein levels of DP isoform‐I in patient pluripotent stem cell‐derived cardiomyocytes. 2 controls (*n* = 4), 1 patient of each genotype (*n* = 6 vs. 4); *p < 0.05 and **p < 0.01 (1‐way ANOVA, Tukey's multiple comparison compared with control keratinocytes); **p < 0.01 (1‐way ANOVA, Tukey's multiple comparison compared with control cardiomyocytes). In the bars, the average percentages of available proteins are depicted. (B) IFM of antigen mapping to DP‐I on *ex vivo* skin in comparison with the *in vitro* cultured keratinocytes and hiPSC‐derived cardiomyocytes. Scale bars = 50 µm. (C) IFM of antigen mapping of N and C‐terminal directed antibodies of DP‐I&II on *ex vivo* skin in comparison with the *in vitro* cultured keratinocytes and hiPSC‐derived cardiomyocytes. Scale bars = 50 µm. The population‐based, quantified IFM intensity at the membrane and cytosol, is depicted in Figure S1D. *The data on hiPSC*‐*derived cardiomyocytes are adapted from Bliley & Vermeer* et al., *and updated with additional patient data from a heterozygous carrier*

Variant *DSP*:c.6687delA resides in *DSP*'s last exon 24 and is predicted to result in a truncated protein (Arg2229Serfs*32) through bypass of nonsense‐mediated mRNA decay (NMD),^12^ while variant *DSP*:c.273+5G>A is predicted to modify the exon 2 splice site. This splice‐site variant is detected in 0.05% of a healthy population^13^ and was once published as part of a cohort related to non‐syndromic arrhythmogenic cardiomyopathy.^10^ Despite the above‐mentioned predictions, both keratinocytes and cardiomyocytes (Figure S1A) of heterozygous *DSP* ^WT/c.6687delA^ carriers showed 50% reduction of DPI and DPII levels, while compound heterozygous *DSP* ^c.273+5G>A/c.6687delA^ carriers showed 70% reduction^9^ (*p* < 0.05) (Figure 2A). In addition, cells with c.6687delA in heterozygous and compound heterozygous state showed a twofold reduction in total *DSP* mRNA levels (with the exception of heterozygous *DSP* ^WT/c.6687delA^ keratinocytes), suggesting NMD. (Figure S1B–C). Indeed, our previous experiments in cardiomyocytes showed that Arg2229Serfs*32 was only detected after NMD inhibition.^9^ However, as total mRNA levels are not reduced in every cell type, this transcript may occasionally skip NMD, but not protein degradation. Moreover, variant c.273+5G>A caused reduced native protein levels, normal mRNA levels, and only normally spliced transcripts in both cardiomyocytes and keratinocytes. However, cloning and analysis of c.273+5G>A into a HEK293 cell‐based minigene splicing assay,^14^, ^15^ a commonly used technique for functional analysis of splicing,^16^, ^17^ was able to show alternatively spliced transcripts (Figure S2). These were the result of intron 2 retention by use of at least two alternative intronic splice sites. These transcripts were nonetheless predicted to result in frame shifts and premature stop‐codons (PTC), leading to either NMD or instable protein degradation. Since this variant is capable of causing alternatively spliced transcripts, in addition to normally spliced transcripts, this would explain the additional 20% DP reduction in *DSP* ^c.273+5G>A/c.6687delA^ carriers compared with *DSP* ^WT/c.6687delA^ carriers. Most strikingly, while DP is clearly reduced on quantitative Western blot in cultured keratinocytes, the expression on immunofluorescence microscopy (IFM) in cultured keratinocytes as well as non‐palmoplantar skin biopsies was undistinguishable from control (Figure 2B–C and Figure S3).

### 4.3 Assessment of intercellular contact and differentiation behaviour of patient keratinocytes

**FIGURE 3:**
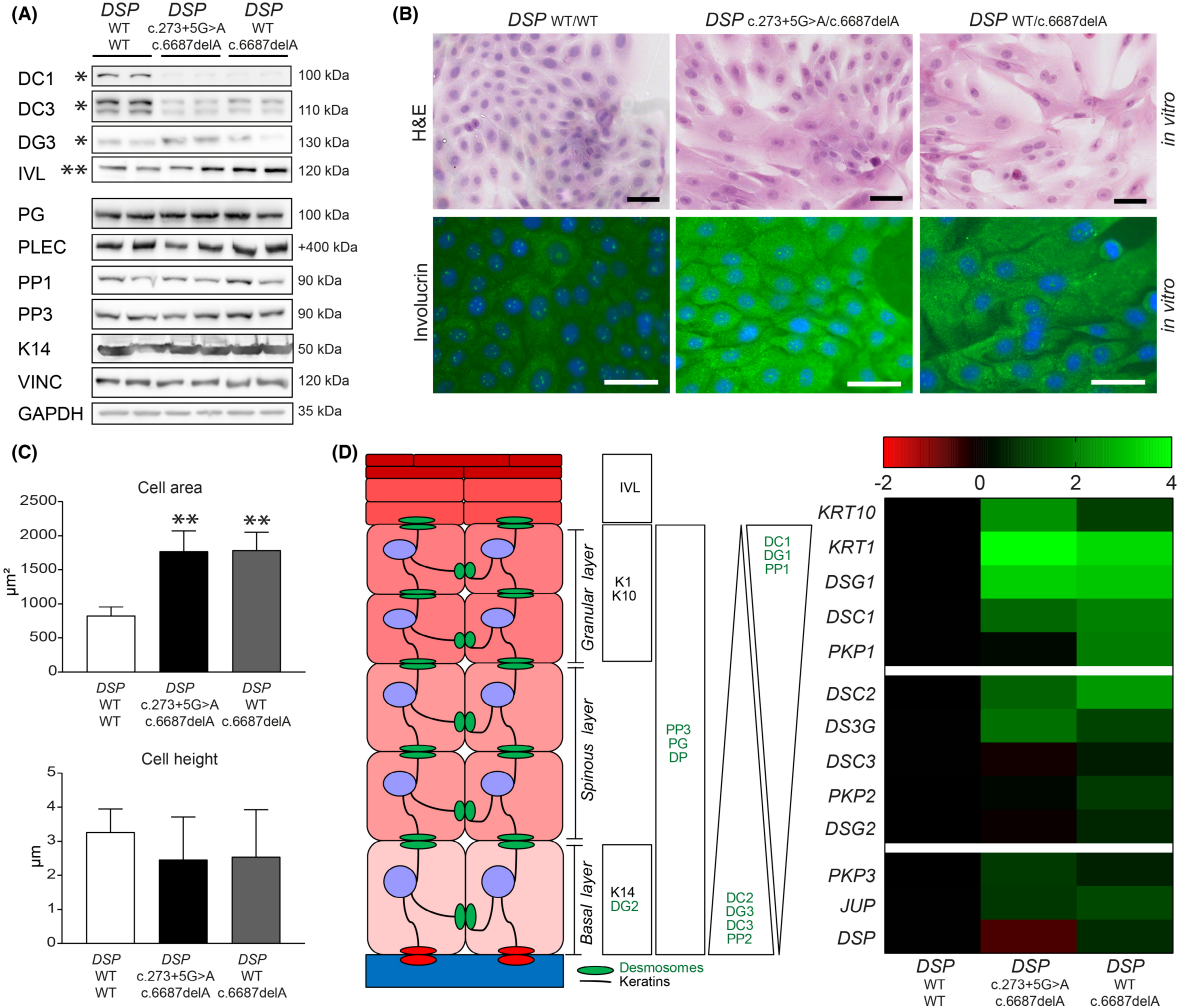
Assessment of patient keratinocytes in culture. (A) Western blots depicting desmocollin‐1 (DC1), desmocollin‐3 (DC3), desmoglein‐3 (DG3), involucrin (IVL), plakoglobin (PG), plectin (PLEC), plakophilin‐1 (PP1), plakophilin‐3 (PP3), keratin 14 (LL002) and housekeeping proteins vinculin (VINC) and GAPDH in keratinocytes of compound heterozygous *(DSP* ^c.273+5G>A/c.6687delA^) and heterozygous (*DSP* ^WT/c.6687delA^) carriers compared with control keratinocytes. Quantification graphs (*n* = 3) can be found in Figure S41A. (B) H&E staining and IFM staining for involucrin on cultured keratinocytes. Scale bars =50 µm. The population‐based, quantified involucrin IFM intensity in the cytosol is depicted in Figure S4B. (C) Quantified the cell area (µm^2^) and cell height (µm) of keratinocytes; **p < 0.01 (one‐way ANOVA, Tukey's multiple comparison compared with control). (D) On the left, a schematic overview of epidermal differentiation and on the right a heatmap of the differential gene expression in patient keratinocytes is depicted. Expression levels are log2(FC)(*n* = 3); plakophilin‐1 (PP2, gene *PKP1*); plakophilin‐2 (PP2, gene *PKP2*); plakophilin‐3 (PP3, gene *PKP3*); desmoglein‐1 (DG1, gene *DSG1*); desmoglein‐2 (DG2, gene *DSG2*); desmoglein‐3 (DG3, gene *DSG3*); desmocollin‐1 (DC1, gene *DSC1*); desmocollin‐2 (DC2, gene *DSC2*); desmocollin‐3 (DC3, gene *DSC3*); plakoglobin (PG, gene *JUP*); keratin 1 (K1, gene *KRT1*); keratin 10 (K10, gene *KRT10*); and keratin 14 (K14)

**FIGURE 4:**
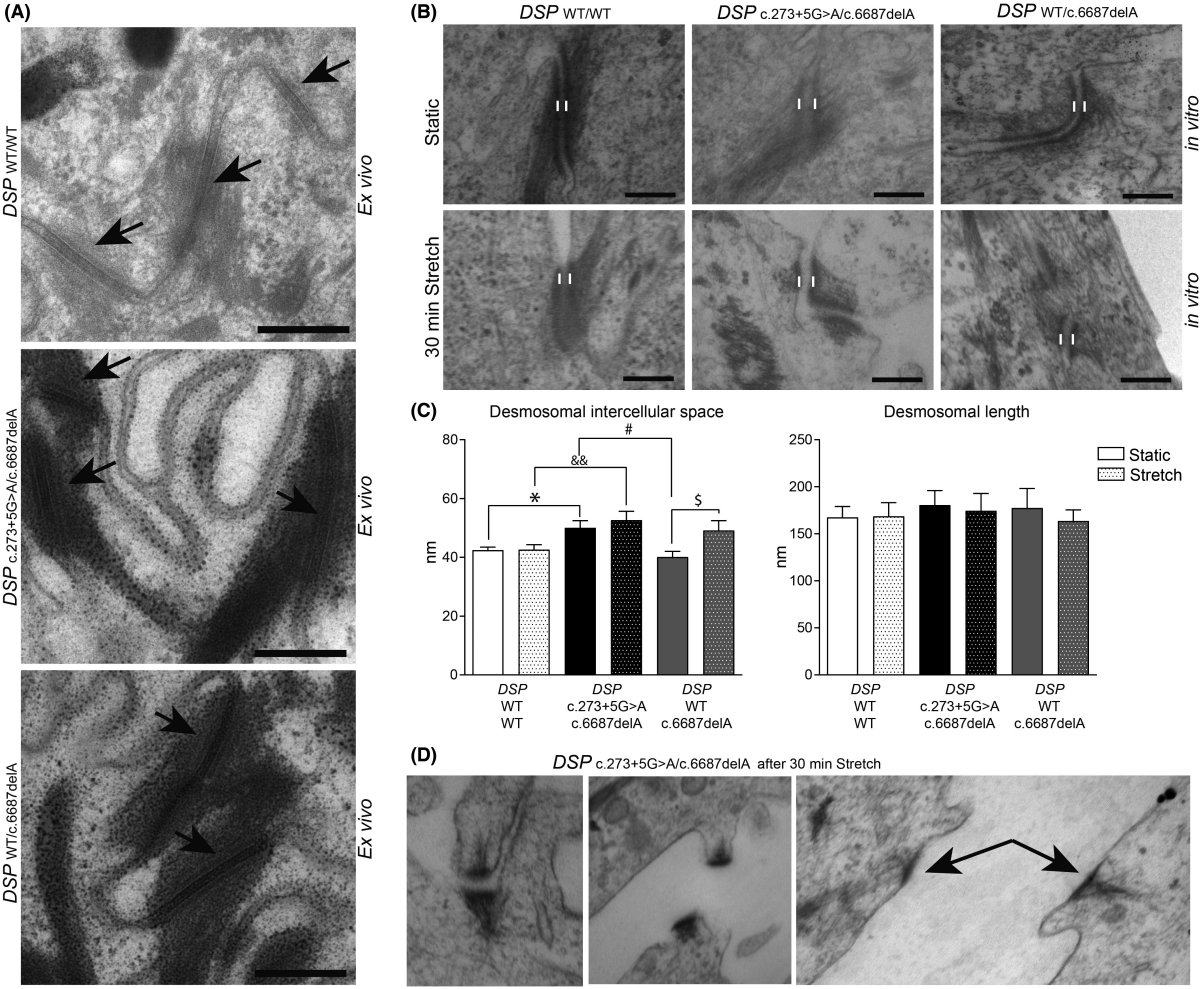
Ultrastructure of desmosomes in *ex vivo* skin and *in vitro* keratinocytes. (A) Examples of desmosomes (arrows), showing normal ultrastructure, in skin biopsies of compound heterozygous *(DSP* ^c.273+5G>A/c.6687delA^) and heterozygous (*DSP* ^WT/c.6687delA^) carriers. Scale bars =200 nm. (B) The ultrastructure of desmosomes in keratinocytes at baseline culture (static) and after stretched condition. Scale bars are 100 nm. (C) Ultrastructure analysis of the desmosomal intercellular space (<100 nm) and desmosomal length, shown as mean ± SEM. $*p* < 0.05 (2‐way ANOVA, Bonferroni's multiple comparison of heterozygous static vs. stretch keratinocytes, *n* = 26 vs. 30); *p < 0,05 (2‐way ANOVA, Bonferroni's multiple comparison of compound heterozygous static vs. control static keratinocytes, *n* = 31 vs. 44 desmosomes); #*p* < 0,05 (2‐way ANOVA, Bonferroni's multiple comparison of heterozygous static vs. compound heterozygous static keratinocytes, *n* = 26 vs. 31); &&*p* < 0.01 (2‐way ANOVA, Bonferroni's multiple comparison compound heterozygous stretch vs. control stretch keratinocytes, *n* = 36 vs. 30). (D) Uncoupled desmosomes (>100 nm) were observed in 5%–10% of the total amount of counted desmosomes after stretch in cultured keratinocytes of the compound heterozygous carrier only

To assess the effects of DP deficiency on the intercellular contact and differentiation behaviour of keratinocytes, we performed a keratinocyte dissociation assay (KDA)^18^ and applied cyclic stretch to 2D cultured keratinocytes of carriers with *DSP* ^WT/c.6687delA^ and *DSP* ^c.273+5G>A/c.6687delA^ variants.^19^ At baseline, the transmembrane cadherins desmocollin‐1 and 3 were twofold to threefold reduced (*p* < 0.05) in both carrier types, whereas *DSP* ^c.273+5G>A/c.6687delA^ keratinocytes also had twofold higher desmoglein‐3 levels (*p* < 0.05) (Figure 3A and Figure S4). Keratinocytes of both carrier types appeared to be enlarged in culture (*p* < 0.01), and mRNA and protein analysis showed a more differentiated state at the same time‐point compared with control^20^ (Figure 3A–D). Yet, electron microscopic analysis of skin biopsies did not emulate any of the aforementioned *in vitro* features, as the ultrastructure appeared normal in both carrier types (Figure 4A). Nonetheless *in vitro*, widened desmosomal intercellular spaces were observed in *DSP* ^c.273+5G>A/c.6687delA^ keratinocytes (*p* < 0.05), and cyclic stretch increased the desmosomal intercellular spaces in *DSP* ^WT/c.6687delA^ keratinocytes (*p* < 0.05) (Figure 4B–C). *DSP* ^c.273+5G>A/c.6687delA^ keratinocytes even occasionally lost cell–cell contact upon cyclic stretch and desmosomes appeared disrupted in 5–10% of the cases (Figure 4D). These changes were not aberrant enough to cause monolayer fragmentations after a KDA (Figure S5A). Moreover, no desmosomal protein upregulations were observed upon stretch other than plakoglobin in control cells (Figure S5B–C) as observed in an earlier study.^19^

## 5 CONCLUSIONS AND PERSPECTIVES

Here, we report a large family in which two *DSP* variants (c.6687delA and c.273+5G>A) segregated with cardiomyopathy, mild PPK and WH. These variants had pronounced effects on both transcription and translation of DPI and DPII isoforms. Contrarily to what was predicted, we reveal that transcripts containing the c.6687delA nonsense variant in the last exon of *DSP* are targeted by NMD and/or instable protein degradation and cause loss of protein from the affected allele. The c.273+5G>A splice‐site variant leads to normal and alternatively spliced transcripts, of which the latter do not result in detectable RNA and protein in patient‐derived cells. In both patients' keratinocytes and cardiomyocytes, compound heterozygosity and heterozygosity for *DSP*:c.6687delA alone resulted in 70% and 50% reduction of DP protein, respectively. Cardiomyopathy was lethal in all compound heterozygous carriers, while milder and not fully penetrant in heterozygous carriers. It remains unknown whether the predicted 20% DP reduction of the heterozygous c.273+5G>A variant alone causes a heart or skin phenotype in this family. Screening of this variant in a cardiomyopathy cohort identified 11 additional carriers from 8 other families. These data combined with our data strongly suggests that the c.273+5G>A variant poses a risk factor for cardiomyopathy and aggravates the phenotype in combination with another pathogenic *DSP* variant.

Literature assessment of *DSP* variants identified 6 other functionally investigated variants that cause various degrees of DP protein deficiency. A 50% reduction of DPI and 50% reduction of DPII mostly associated with a moderate form of cardiomyopathy, with involvement of PPK based on data from dominantly inherited variants c.699G>A: p.(Trp233*),^21^ c.939+1G>A: p.(Gln331*)^22^, ^23^, ^24^, ^25^ and c.1348C>G: p.(Arg451Gly).^26^ Variants in the ROD domain of DPI, that cause 50% reduction of DPI in heterozygous state (c.3805C>T: p.(Arg1269*)^27^) and 100% reduction of DPI in homozygous state (c.3799C>T: p.(Arg1267*),^5^ while leaving DPII intact, cause a moderate form of cardiomyopathy due to the dominant variant, while very severe and early onset cardiomyopathy due to the recessive variant. Although DPI is the main isoform in the heart, the cardiac phenotype of these patients suggests that DPII may be more important in the heart than initially assumed, as full loss of DP unlikely allows human heart formation.^28^ In the skin, loss of DPI also caused a more severe skin phenotype, observed as epidermolytic PPK with WH syndrome. Moreover, a homozygous state of in‐frame deletion variant c.969_974del; p.(Lys324_Glu325del) causes PPK, WH and cardiomyopathy, and caused significantly more than 50% reduction of both DPI and DPII isoforms. These findings support our data, but additionally need to be confirmed by more experimentally investigated *DSP* variants. Intriguingly, no other functionally assessed variants that lead to a premature termination‐codon in exon 24 have been reported to allow NMD targeting. Moreover, only one other splice site variant (*DSP*:c.939+1G>A) in intron 7 has been functionally investigated in patients.^23^, ^24^, ^25^ Currently, much is unknown about the pathophysiology of splice‐site variants in *DSP*, as thus far only two have been fully investigated, while our reported variant certainly adds a new level of genetic complexity.

In retrospect, our dynamic loading platform of induced pluripotent stem cell (hiPSC)‐derived engineered heart tissues has recently been successful in mimicking key characteristics of desmosome‐linked *in vivo* disease,^9^ as well as patients with intermediate filament‐linked disease,^29^ both of which cause skin disease.^30^, ^31^ While *in vitro* investigation of patient keratinocytes in this study showed altered differentiation, reflecting the PPK, other findings did not mimic the mild features seen in patients. The differences between the *in vivo* skin situation and the *in vitro* characteristics of cultured keratinocytes from the same site may be caused by the extra demands on keratinocytes in culture and/or the lack of compensatory mechanisms present in the *in vivo* situation but lacking in *in vitro* culturing conditions. In that sense, three‐dimensional skin models,^32^ derived from patient primary or hiPSC‐derived keratinocytes,^32^, ^33^, ^34^ could better mimic the situation of *in vivo* disease. Nonetheless, contrary to the seemingly normal DP protein intensity on IFM in both non‐palmoplantar skin and cultured keratinocytes of the same site, protein levels in cultured keratinocytes on blot showed significant DPI & II deficiency, reduction of desmocollin‐1 and desmocollin‐3, altered differentiation and impaired cell–cell contacts, without cell‐sheet dissociation after a KDA. These results are in line with gradual depletion of DPI and /or DPII using RNA interference in HaCaT‐keratinocytes. Reduction in total DPI & II expression reduced the amounts of plakophilin‐1, desmocollin‐2 and desmocollin‐3,^19^, ^35^ while DPI had a greater influence on the expression levels of desmocollin‐3 than DPII. These results also suggest that the two DP splice variants are not completely redundant in function, as DPII was the key component of intermediate filament stability and desmosome‐mediated adhesion in skin‐like cells.^19^ Furthermore, cell‐sheet dissociation did not seem to correlate with DPI depletion (90% reduction in HaCaT, nor the 70% reduction reported in this study), but more so with DPII depletion (90% reduction in HaCaT, but not with the 70% reduction reported in this study). These data and ours suggest that threshold levels of DPII to induce fragmentation lie somewhere between 10% and 30% of wildtype expression. Our results further indicate that IFM expression and EM of *ex vivo* non‐palmoplantar skin are not reliable in indicating the molecular defect, let alone to predict cardiomyopathy later in life.

Many studies have attempted to establish a genotype–phenotype correlation for *DSP* variants. However, the effect of the vast majority of reported variants has been predicted instead of being experimentally assessed. Our data point out that the actual effect of *DSP* nonsense and splice‐site variants on DP protein is more complex than current *in silico* programs can predict. The unique pathogenicity of the variants reported here has broadened the understanding of the *DSP* genotype–phenotype correlation in both skin and heart and should be taken into account when evaluating other nonsense or splice‐site variants. In addition, the particular pattern of inheritance, with either simultaneous or separately segregating DSP variants within the same family, hereby strongly supports the theory of a dose‐dependent disease severity in both the skin and heart. Analysis of variants for the effect on RNA and protein level and tissue effects in both skin and heart, preferably in 3D models, will be the stepping stone to elucidate the pathogenicity of *DSP* variants and better genotype–phenotype correlations in the future. Above all, these are essential in the light of advancements in more targeted protein, RNA and/or DNA based therapies.

## CONFLICT OF INTEREST

The authors have nothing to declare.

## AUTHOR CONTRIBUTIONS

MCSCV, PvdM and MCB conceptualized the study. MCSCV, DA, DK, AMN, YMH, HW and JDHJ were involved in the data curation. MCSCV prepared the draft and wrote the manuscript. MCB, PvdM, HHP, HHWS and MPvdB reviewed and edited the manuscript. All authors have read and approved the final manuscript.

## Supporting information


**FIGURE S1** Additional hiPSC characterization and *DSP* mRNA levels in hiPSC‐derived cardiomyocytes and primary keratinocytes. (A) On the left, the karyotypes from two hiPSC‐lined derived from patient III:4 are depicted, where the expression of pluripotency markers, OCT3/4 and SSEA‐4, was determined in one of these lines, using IFM staining. After differentiation to cardiomyocytes, cells express cardiac‐specific Troponin T and slow skeletal Troponin I. Scale bars upper panel =100 µm, lower panels 30 µm. (B) mRNA levels of *DSP* isoform‐I in patient pluripotent stem cell‐derived cardiomyocytes. 2 control donor included (*n* = 5), 1 patient of each genotype, derived from 2 hiPSC‐lines each (*n* = 5); **p < 0.01 (1‐way ANOVA, Tukey's multiple comparison compared to control cardiomyocytes). (C) mRNA levels of *DSP* isoform‐I or I&II combined in cultured primary keratinocytes. 2 controls (*n* = 5), 1 patient of each genotype (*n* = 6/patient); #*p* < 0.05 (1‐way ANOVA, Tukey's multiple comparison between compound vs. heterozygous patient keratinocytes). (D) IFM intensity of different DP antibodies, quantified at the membrane and at the cytosol. ***p < 0.05 (2‐way ANOVA, Tukey's multiple comparison between control vs. compound heterozygous patient keratinocytes). #*p* < 0.05 (2‐way ANOVA, Tukey's multiple comparison between compound heterozygous vs. heterozygous patient keratinocytes). *Panel B is adapted from Bliley & Vermeer* et al., *and updated with additional patient data from a heterozygous carrier (III:4)*.
**FIGURE S2** Results from the minigene splicing assay (c.273+5G>A). (A‐B) Results from transfection of *DSP* wild type and variant c.273+5G>A minigene constructs into HEK293 cells showed multiple alternative splice transcripts that included: (A) a product with over 250 base pair retention of intron 2 (total) leading to longer transcripts (no new donor site predicted on HSF) and (B) a product with a 61 base pair (partial) intron 2 retention (as predicted by HSF finder with consensus value 88.05). The position at the arrows indicate the wild type and variant into the sequence. Both products were confirmed with Sanger sequencing as indicated. Note that for alternative splicing product 1 + 2, only one sequencing result is shown. Alternative splicing products 0, 3 and 5 are products present with too low concentrations for Sanger sequencing.
**FIGURE S3** Localization of key proteins specific for epidermal layers in *ex vivo* non‐palmoplantar skin biopsies. IFM of involucrin (IVL) specific for the stratum corneum, desmoglein‐1 (DG1), desmocollin‐1 (DC1) mostly expressed in the suprabasal layer, plakoglobin (PG), desmocollin‐3 (DC3), desmoglein‐3 (DG3), and intermediate filament protein keratin 14 (K14) specific for the basal layer, in *ex vivo* skin biopsies. Scale bars =50 µm.
**FIGURE S4** Desmosomal protein quantification and localization in cultured keratinocytes. (A) Total protein expression of desmocollin‐1 (DC1), desmocollin‐3 (DC3), desmoglein‐3 (DG3), involucrin (IVL), plakoglobin (PG), plectin (PLEC), plakophilin‐1 (PP1), plakophilin‐3 (PP3) and keratin 14 (K14) in cultured keratinocytes determined by western blot; *p < 0.05 (1‐way ANOVA, Tukey's multiple comparison compared to control keratinocytes); **p < 0.01 (1‐way ANOVA, Tukey's multiple comparison compared to control keratinocytes); ***p < 0.001 (1‐way ANOVA, Tukey's multiple comparison compared to control keratinocytes); *n* = 3/group. (B) IFM of DC1, DC3, DG3 and PG in cultured keratinocytes. Scale bars =50 µm. In addition, IFM intensity quantified at the membrane and at the cytosol is graphed. *p < 0.05/**p < 0.01/***p < 0.001 (2‐way ANOVA, Tukey's multiple comparison compared to control keratinocytes). #*p* < 0.05/###*p* < 0.001 (2‐way ANOVA, Tukey's multiple comparison between compound heterozygous vs. heterozygous patient keratinocytes). *p < 0.05 (1‐way ANOVA, Tukey's multiple comparison of involucrin in the cytosol compared to control keratinocytes).
**FIGURE S5** Keratinocyte dissociation assay and protein quantification after cyclic stretch. (A) Keratinocyte dissociation assay in cultured keratinocytes (in triplo). (B) Plakoglobin (PG) localization on IFM in static vs. stretch keratinocytes. Scale bars =50 µm. (C) Whole cell protein expression of desmosomal constituents on blot in static vs. stretch keratinocytes, including quantified graphs. *p < 0.05 (unpaired *t*‐test, static vs. stretch control keratinocytes).
**TABLE S1** Carriers for *DSP*:c.273+5G>A.
**TABLE S2** Primers.
**TABLE S3** Antibodies.

## Data Availability

All data can be found in the main manuscript and Appendix S1.
