# Supplementary material for: Functional investigation of two simultaneous or separately segregating DSP variants within a single family supports the theory of a dose‐dependent disease severity
Source: Exp Dermatol. 2022 Apr 1;31(6):970–9. doi: 10.1111/exd.14571 (PMC9322008; doi:10.1111/exd.14571)
Supplement: Supplementary file 1 — FIGURE S1 Additional hiPSC characterization and DSP mRNA levels in hiPSC‐derived cardiomyocytes and primary keratinocytes. (A) On the left, the karyotypes from two hiPSC‐lined derived from patient III:4 are depicted, where the expression of pluripotency markers, OCT3/4 and SSEA‐4, was determined in one of these lines, using IFM staining. After differentiation to cardiomyocytes, cells express cardiac‐specific Troponin T and slow skeletal Troponin I. Scale bars upper panel =100 µm, lower panels 30 µm. (B) mRNA levels of DSP isoform‐I in patient pluripotent stem cell‐derived cardiomyocytes. 2 control donor included (n = 5), 1 patient of each genotype, derived from 2 hiPSC‐lines each (n = 5); **p < 0.01 (1‐way ANOVA, Tukey's multiple comparison compared to control cardiomyocytes). (C) mRNA levels of DSP isoform‐I or I&II combined in cultured primary keratinocytes. 2 controls (n = 5), 1 patient of each genotype (n = 6/patient); #p < 0.05 (1‐way ANOVA, Tukey's multiple comparison between compound vs. heterozygous patient keratinocytes). (D) IFM intensity of different DP antibodies, quantified at the membrane and at the cytosol. ***p < 0.05 (2‐way ANOVA, Tukey's multiple comparison between control vs. compound heterozygous patient keratinocytes). #p < 0.05 (2‐way ANOVA, Tukey's multiple comparison between compound heterozygous vs. heterozygous patient keratinocytes). Panel B is adapted from Bliley & Vermeer et al., and updated with additional patient data from a heterozygous carrier (III:4). FIGURE S2 Results from the minigene splicing assay (c.273+5G>A). (A‐B) Results from transfection of DSP wild type and variant c.273+5G>A minigene constructs into HEK293 cells showed multiple alternative splice transcripts that included: (A) a product with over 250 base pair retention of intron 2 (total) leading to longer transcripts (no new donor site predicted on HSF) and (B) a product with a 61 base pair (partial) intron 2 retention (as predicted by HSF finder with consensus value 88 [file EXD-31-970-s001.docx]

**ONLINE SUPPLEMENTAL MATERIAL**

**Gene panel: inherited cardiomyopathies**

*ABCC9, MYOZ1, MYOZ2, NEXN, PKP2, ACTC1, CALR3, CAV3, DTNA, EMD, EYA1, GLA, GATAD1, JPH2, JUP, LAMA4, LAMP2, LDB3, LMNA, MYBPC3, MYH6, DMD, DSC2, DSG2, DSP, MYL2, MYL3, MYPN, PLN, ACTN2, ANKRD1, BAG3, PRKAG2, SGCD, MYH7, CRYAB, CSRP3, DES, TMEM43, TNNC1, TNNI3, TNNT2, TPM1, TTN, RBM20, RYR2, SCN5A, TTR, TXNRD2, SOD2, TAZ, TBX20, TCAP, VCL* (54 genes).

**Primary keratinocyte culture and cyclic stretch**

All primary keratinocyte cultures were expanded and passaged in 0.2 mM calcium containing CnT-PRIME (CELLNTEC) medium and regularly checked for mycoplasma contamination. For experiments, keratinocytes were seeded to stretchable plates (Bioflex® plates collagen I) and grown in 1.2 mM calcium containing media (CnT-PR-D) for 4 days until 50% confluence. To induce mechanical stretch, cells were subject to 30 min of 15% stretch at 3Hz, before harvesting RNA and protein. *Of note, hiPSC-derived cardiomyocytes were cultured and analyzed according to our previous paper.*

**Keratinocyte dissociation assay (KDA)**

Keratinocytes were seeded on 12-well plates and cultured in 0.2 mM calcium until 100% confluency (day 0). Cells were maintained in low calcium until day 6. At day 6, cells were switched to CnT-PR-D medium with low calcium, At day 7, cells were incubated with CnT-PR-D medium with 1.2 mM high calcium for 24hrs. On day 8, a keratinocyte dissociation assay (KDA) was performed. Prior to KDA, a 1 cm was cut from the tip-ending of a 1000µl pipette tip and the tip was coated in 1% BSA/PBS solution. Cells were washed 2x with PBS and incubated with 400 ul dispase (2.4 U/L) until monolayers (sheets) detached. Sheets were gently washed with PBS, to remove residual dispase. Then, 1 ml PBS was added to each well and with the cutted-tip, sheets were resuspended 10 times, by pipetting up and down at similar speed. To prevent the sheets from further breakage, 80 µl of formaldehyde (37%) was added to the well. A crystal violet solution was added to color-contrast the dissociated cell clumps/ sheets. Hereinafter, pictures were taken and cell clumps/sheets were counted when dissociation occurred.

**HEK293 *in vitro* minigene splicing assay**.

Genomic wild type and *DSP*:c.273+5G>A fragments, containing exon(s) in the region of interest and up to 250 bp of 5’and 3’ flanking intronic sequences, were PCR-amplified **(Table S2).** These products were subcloned into the pJET cloning vector, following manufacturer’s instructions (Thermo Fisher). Correct inserts were cloned into the pSPL3 exon-trapping vector (Invitrogen). HEK293 cells were plated in 6-well plates and cultured in regular DMEM with glutamine, 10% FCS and 1% P/S. After 24 hours, cells were transfected with 1 µg plasmid DNA using polyethylenimine (Polyscience INC). The empty pSPL3 vector was used as negative control. All experiments were performed in duplicates and after 48 hours, RNA was isolated.

**PCR and Sanger sequencing**

To calculate the relative expression of genes with the ∆∆-Ct method, RNA from primary keratinocytes were isolated using TRIzol (Sigma). cDNA was synthesized by reverse transcription (RevertAid H Minus First Strand cDNA Synthesis Kit, Thermo Fisher) and real-time PCR using SYBR™ Green was performed **(Table S2,** primers that detect total gene expression). In addition, a real-time PCR was also performed to amplify the mutation sites in *DSP* with flanking sequence **(Table S2).** In parallel, RNA from transfected HEK293 cells was used as a template to synthesize cDNA using the cDNA random hexamer primers pd(N)6 and/or oligo (dT)18 primers. PCR was performed using primers, complementary to the sequences of exons standardly available in pSL3, **(Table S2)** with Amplitaq Gold Fast PCR mix (Thermo Fisher) and the following amplification program: 5 min at 96°C, followed by 35 cycles of 1 min at 94°C, 1 min at 58°C, 1 min at 72°C and a final elongation time of 10 mins at 72°C. The specific *DSP* PCR products amplified from primary keratinocytes and transfected HEK293 cells, were analyzed using 2% agarose gel electrophoresis. The amplified PCR products were purified using EXOSAP IT (GE Healthcare) and sequenced (BigDye V3.1 Terminator chemistry; Applied Biosystems) Reaction products were run on the ABI3730XL Genetic Analyzer (Applied Biosystems) and sequences were evaluated using Mutation Surveyor software (SoftGenetics LCC).

***In silico* predictions**

*DSP*:c.273+5G>A> The Human Splicing Finder 3.1, SSF and MaxEntScan in silico algorithms predicted this variation to have an impact on splicing by complete abolition of the natural donor 5’ splice site of exon 2, while the GeneSplicer score was reduced by more than 50% between wild type and c.273+5G>A mutant. To predict the functional consequences of this variant after aberrant splicing in HEK293 transfection, Alamut® software version 2.11 and Interactive Bio software were used. *DSP*:c.6687delA> Variant *DSP*:c.6687delA is located in the last exon, which results in a frameshift followed by a premature stop-codon [p.(Arg2229serfs*32)] As NMD in mammalian cells is linked to pre-mRNA splicing, lack of an upstream intron therefore predicts this transcript to bypass NMD.

**Gel electrophoresis and western blotting**

Cellular proteins were extracted using a buffer containing 62 mM Tris-HCl, 2,5% SDS and 1 mM EDTA, protease inhibitor (Roche 11873580001), phosphatase inhibitor cocktail 3 (p2850; Sigma) and sodium orthovanadate. Before loading, sample buffer was added, for the total extract to also contain 10% glycerol, 5% β-mercaptoethanol and bromophenol blue. Proteins were separated in SDS-PAGE gels and transferred to PVDF or nitrocellulose membranes. Membranes were blocked and incubated with primary **(Table S3)** and secondary HRP-labeled antibodies, before detection with electrochemiluminescence.

**Immunofluorescence microscopy (IFM)**

Cryo-frozen skin tissues were sectioned to histology slices with a cryostat microtome and dried for 30min. Histology slices were then incubated with primary antibody **(Table S3)** in 1% OVA/PBS for 30min at RT. Histology slices were then washed and incubated with secondary antibody in 1% OVA/PBS for 30min at RT. The slices were then washed and mounted with DAPI. Keratinocyte cultures were fixated for 5min with -20 ºC methanol: aceton(1:1). Fixated cells were blocked for 30min with blocking buffer containing 3%BSA/PBS and 2% serum (host of secondary antibody). Cells were incubated with primary **(Table S3)** antibody in blocking buffer for 1hr at RT. Cells were washed and subsequently incubated with secondary antibody in blocking buffer for 1hr at RT. Hereinafter, cells were washed and mounted with mounting media containing DAPI. All slides were imaged using a Leica DMI6000B fluorescent microscope.

**Protein localization on IFM**

The abundance of protein signal at the membrane and cytosol was measured in 40x magnified images. Using FIJI, the total pixel intensity was plotted against the pixel count. Then the following parameters were determined separately: [1] the mean of the maximal intensity pixels in the cytosol, [2] the total number of nuclei in the image, [3] the mean pixel intensity in the cytosol, [4] and the mean pixel intensity at the membrane. The mean pixel intensity in the cytosol was used for the cytosolic fraction. To determine the membrane fraction, the mean of the maximal intensity pixels at the cytosol [1] was used as a cut-off to determine the number of pixels at the membrane as a percentage of the total number of pixels. This number was normalized for the number of nuclei [2]. This was subsequently normalized against the mean pixel intensity measured in the cytosol [3] and normalized for the mean of the intensity of pixels at the membrane [4]. For each experimental set, the fold change was calculated compared to the abundance of signal measured in control cells.

**Electron microscopy**

Skin biopsies and static and stretched keratinocytes were fixed with a 2%glutaraldehyde/2%formaldehyde mixture in 0,1M sodium cacodylate at 4°C. After post-fixation in 1%osmium tetroxide/1,5%potassium ferrocyanide (2 hours at 4°C), cells/tissues were dehydrated using ethanol and embedded in EPON epoxy resin. 60nm sections were cut transverse in cell direction and contrasted using 2% uranylacetate in water for 45min followed by Reynolds lead citrate for 1min. Images of desmosomes were taken with a CM100 TEM microscope at a 46.000x magnification and FIJI was used to make intensity plots. A proxy of the desmosomal intercellular space was calculated in cultured keratinocytes. This defines the length between the two highest intensity peaks (located approximately within both membranes/outer dense plaques). This distance is larger in cultured cells than the space measured in the skin. All desmosomal intercellular spaces up to 100 nm were included in the statistical analysis. Up to 40 desmosomes were measured in each group. Spaces larger than 100 nm, were considered as uncoupled desmosomes. The desmosome length was also measured and plotted.

**Statistics**

All data are represented as means ± standard error of the mean (SEM). For all data, a Shapiro-Wilk normality test was performed and outliers were excluded using ROUT (Q=1) A p<0.05 was considered statistically significant. For cellular experiments, differences between patient and control keratinocytes were calculated using an 1-way ANOVA, Tukey’s multiple comparisons test. Difference between cell lines under either static or stretched condition were calculated using a 2-way ANOVA, Bonferroni’s multiple comparisons test. Specifics regarding the used tests are indicated in the respective figure legends. Statistics were performed using GraphPad Prism.

**Data availability statement**

Datasets related to this study are found in the text and supplements.

**SUPPLEMENTARY FIGURES/TABLES**

**
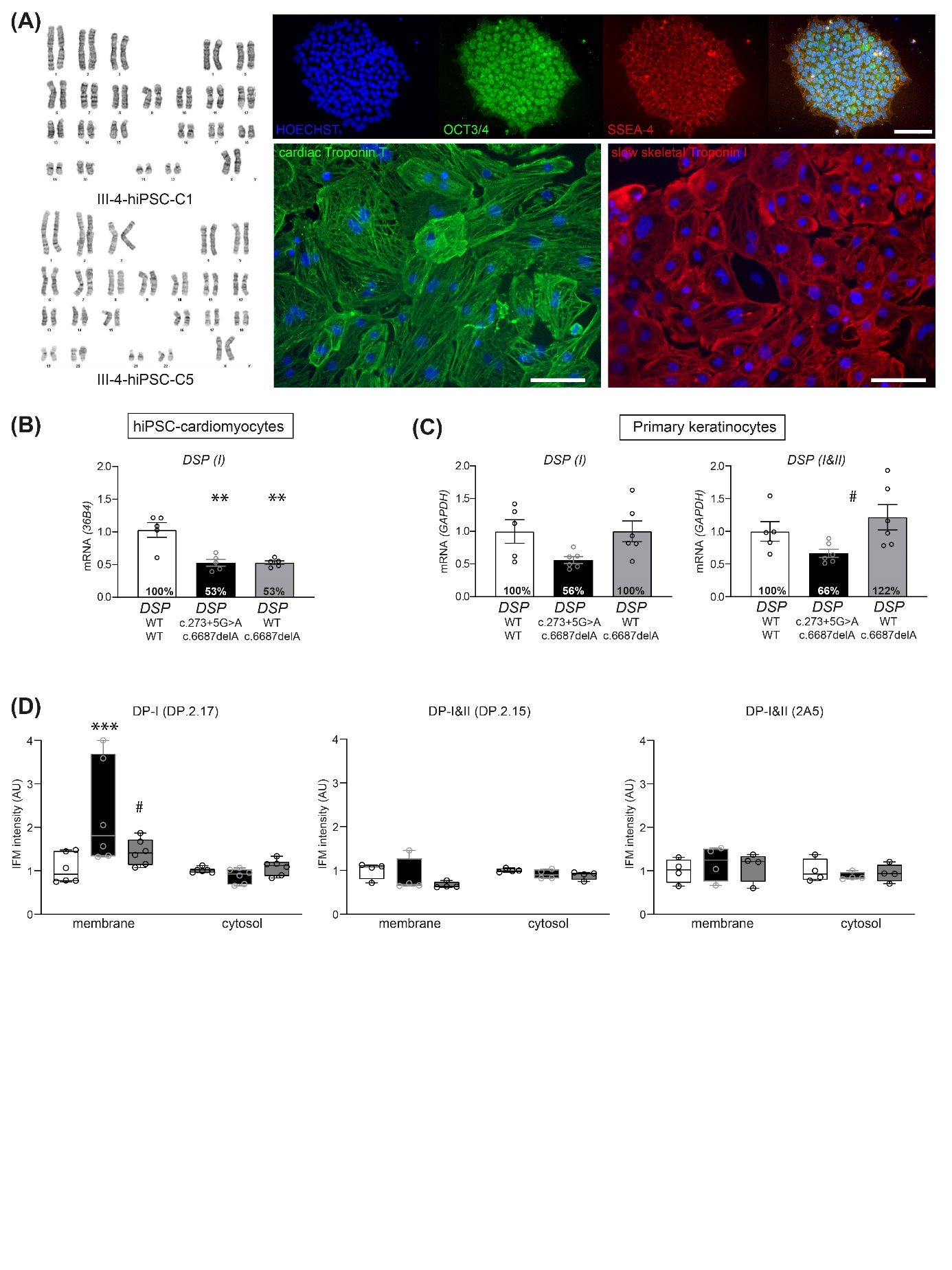
**

**Figure S1: Additional hiPSC characterization and *DSP* mRNA levels in hiPSC-derived cardiomyocytes and primary keratinocytes**

**(A)** On the left, the karyotypes from two hiPSC-lined derived from patient III:4 are depicted, where the expression of pluripotency markers, OCT3/4 and SSEA-4, was determined in one of these lines, using IFM staining. After differentiation to cardiomyocytes, cells express cardiac-specific Troponin T and slow skeletal Troponin I. Scale bars upper panel = 100 µm, lower panels 30 µm. **(B)** mRNA levels of *DSP* isoform-I in patient pluripotent stem cell-derived cardiomyocytes. 2 control donor included (n=5), 1 patient of each genotype, derived from 2 hiPSC-lines each (n=5); **p<0.01 (1-way ANOVA, Tukey’s multiple comparison compared to control cardiomyocytes). **(C)** mRNA levels of *DSP* isoform-I or I&II combined in cultured primary keratinocytes. 2 controls (n=5), 1 patient of each genotype (n=6/patient); #p<0.05 (1-way ANOVA, Tukey’s multiple comparison between compound vs. heterozygous patient keratinocytes). (**D**) IFM intensity of different DP antibodies, quantified at the membrane and at the cytosol. ***p<0.05 (2-way ANOVA, Tukey’s multiple comparison between control vs. compound heterozygous patient keratinocytes). #p<0.05 (2-way ANOVA, Tukey’s multiple comparison between compound heterozygous vs. heterozygous patient keratinocytes). *Panel B is adapted from Bliley & Vermeer et al., and updated with additional patient data from a heterozygous carrier (III:4).*

**
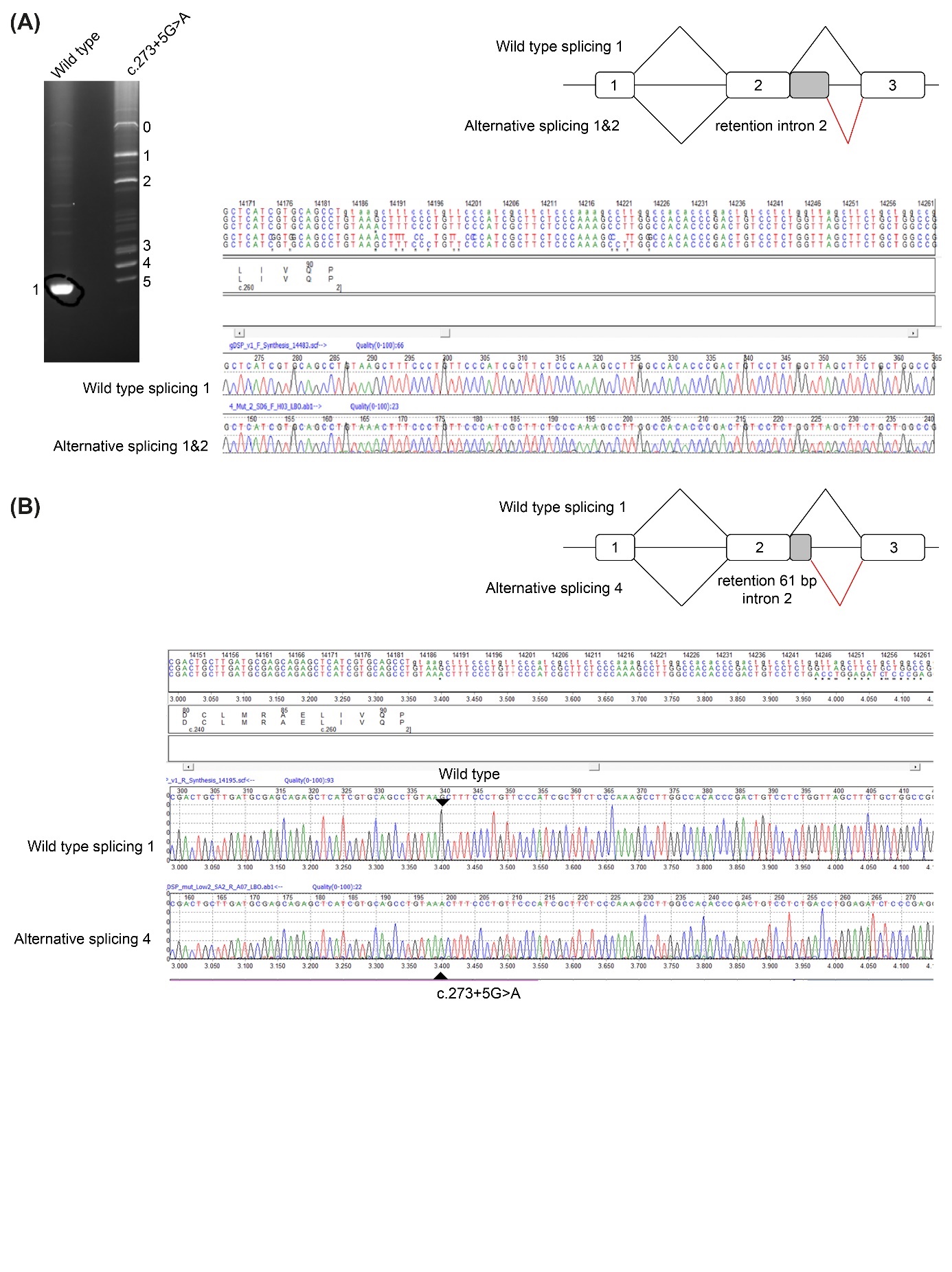
Figure S2: Results from the minigene splicing assay (c.273+5G>A)**

**(A-B)** Results from transfection of *DSP* wild type and variant c.273+5G>A minigene constructs into HEK293 cells showed multiple alternative splice transcripts that included: **(A)** a product with over 250 base pair retention of intron 2 (total) leading to longer transcripts (no new donor site predicted on HSF) and **(B)** a product with a 61 base pair (partial) intron 2 retention (as predicted by HSF finder with consensus value 88.05). The position at the arrows indicate the wild type and variant into the sequence. Both products were confirmed with Sanger sequencing as indicated. Note that for alternative splicing product 1+2, only one sequencing result is shown. Alternative splicing products 0, 3 and 5 are products present with too low concentrations for Sanger sequencing.


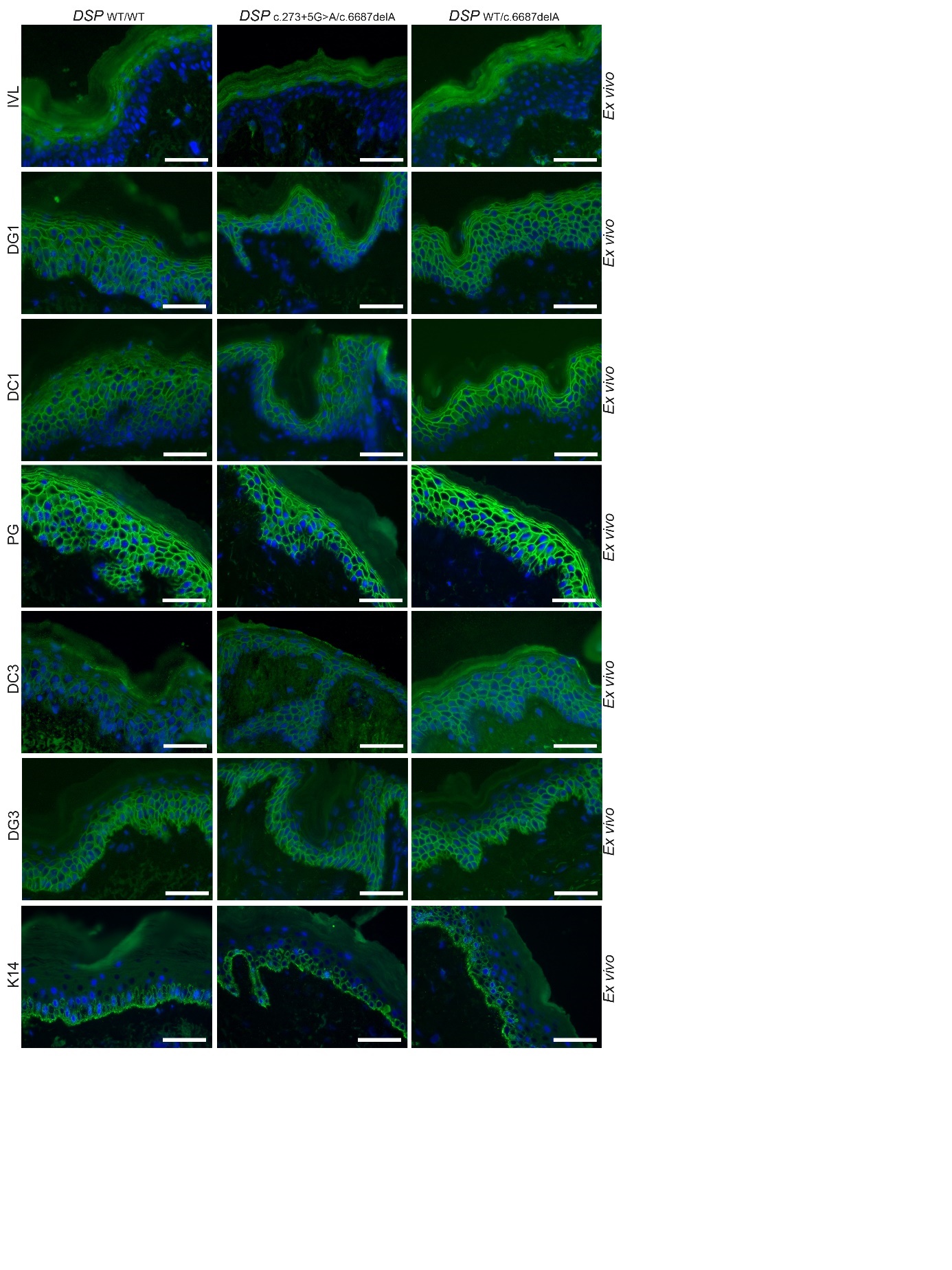
**Figure S3: Localization of key proteins specific for epidermal layers in *ex vivo* non-palmoplantar skin biopsies.**

IFM of involucrin (IVL) specific for the stratum corneum, desmoglein-1 (DG1), desmocollin-1 (DC1) mostly expressed in the suprabasal layer, plakoglobin (PG), desmocollin-3 (DC3), desmoglein-3 (DG3), and intermediate filament protein keratin 14 (K14) specific for the basal layer, in *ex vivo* skin biopsies. Scale bars = 50 µm.

**
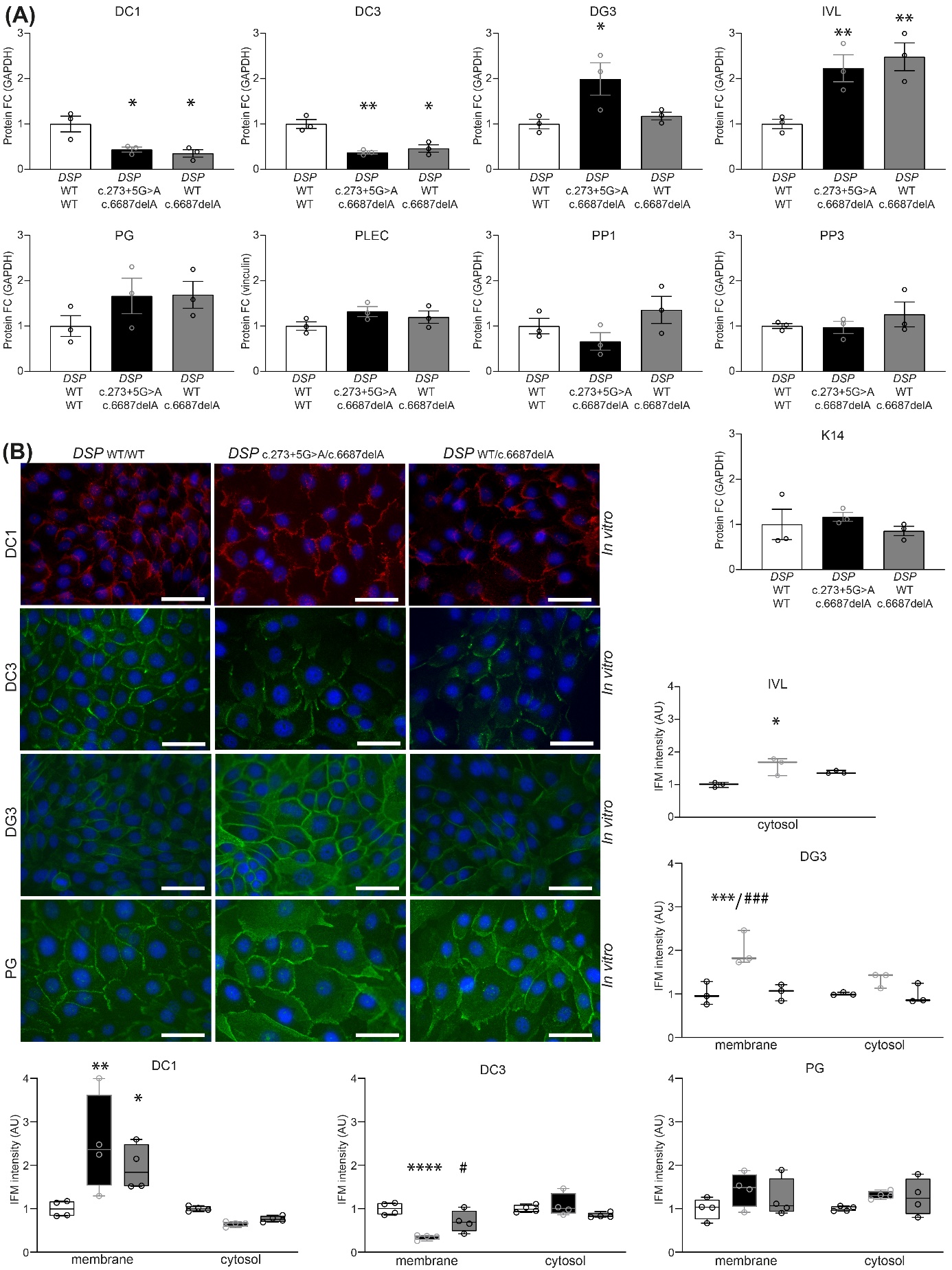
 Figure S4: Desmosomal protein quantification and localization in cultured keratinocytes**

**(A)** Total protein expression of desmocollin-1 (DC1), desmocollin-3 (DC3), desmoglein-3 (DG3), involucrin (IVL), plakoglobin (PG), plectin (PLEC), plakophilin-1 (PP1), plakophilin-3 (PP3) and keratin 14 (K14) in cultured keratinocytes determined by western blot; * *P*<0.05 (1-way ANOVA, Tukey’s multiple comparison compared to control keratinocytes); ** *P*<0.01 (1-way ANOVA, Tukey’s multiple comparison compared to control keratinocytes); *** *P*<0.001 (1-way ANOVA, Tukey’s multiple comparison compared to control keratinocytes); n=3/group. **(B)** IFM of DC1, DC3, DG3 and PG in cultured keratinocytes. Scale bars = 50 µm. In addition, IFM intensity quantified at the membrane and at the cytosol is graphed. *p<0.05/**p<0.01/***p<0.001 (2-way ANOVA, Tukey’s multiple comparison compared to control keratinocytes). #p<0.05/ ###p<0.001 (2-way ANOVA, Tukey’s multiple comparison between compound heterozygous vs. heterozygous patient keratinocytes). *p<0.05 (1-way ANOVA, Tukey’s multiple comparison of involucrin in the cytosol compared to control keratinocytes)

**
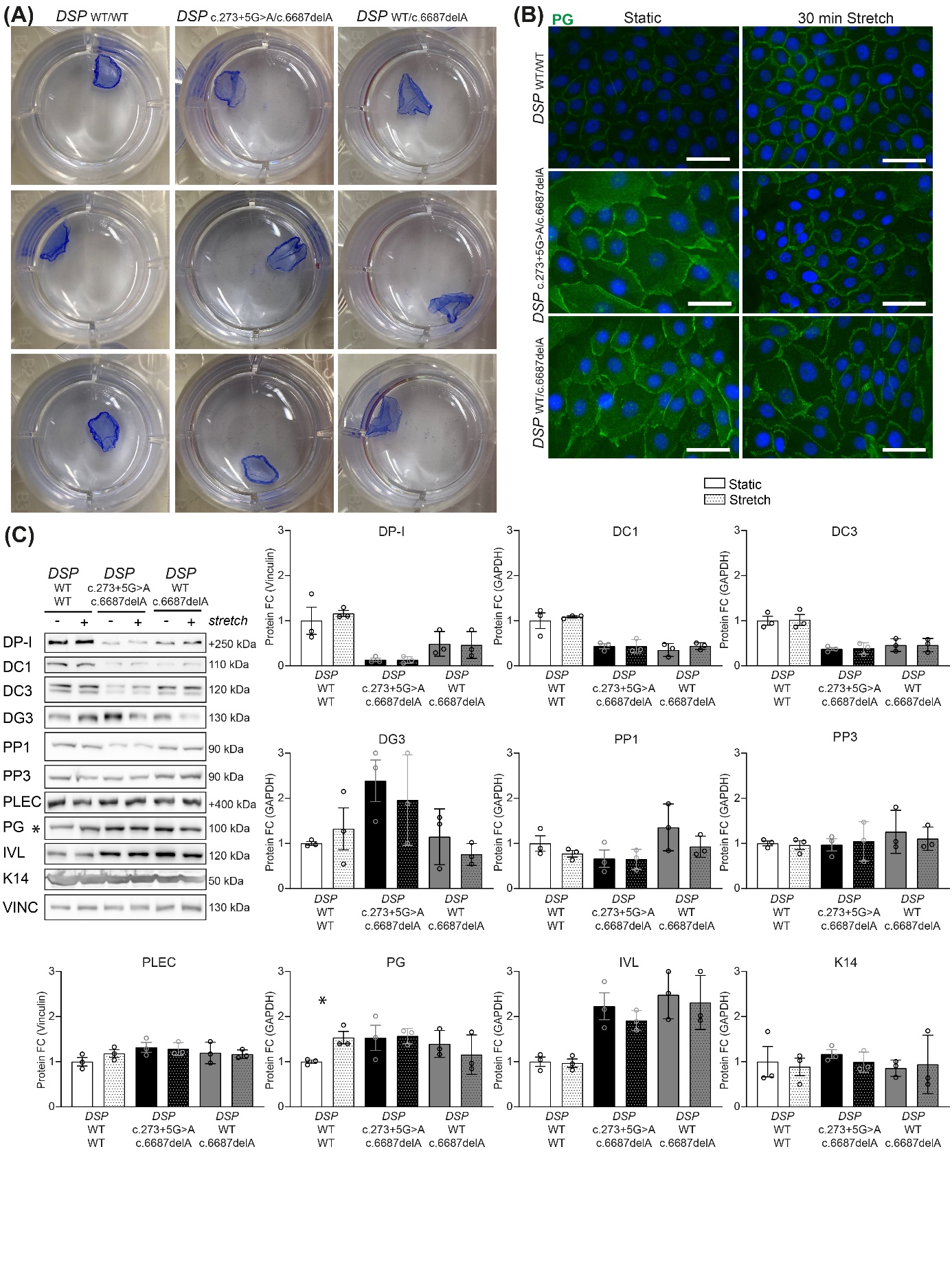
**

**Figure S5: Keratinocyte dissociation assay and protein quantification after cyclic stretch**

**(A)** Keratinocyte dissociation assay in cultured keratinocytes (in triplo). (**B)** Plakoglobin (PG) localization on IFM in static vs. stretch keratinocytes. Scale bars = 50 µm. (**C)** Whole cell protein expression of desmosomal constituents on blot in static vs. stretch keratinocytes, including quantified graphs. * *P*<0.05 (unpaired t-test, static vs. stretch control keratinocytes).

**Table S1: Carriers for *DSP*:c.273+5G>A**

| **Patient no** | **Age** | **Sex** | **Family** | **Phenotype** | **Other variants** |
| --- | --- | --- | --- | --- | --- |
| 1 | 31† | M | This family (1) | Unknown, died young due to unrelated cause |  |
| 2 | 23† | F | This family (1) | WH, PPK, cardiomyopathy | *DSP*:c.6687delA |
| 3 | 52, HTx | F | This family (1) | WH, PPK, cardiomyopathy | *DSP*:c.6687delA |
| 4 | 24 | M | This family (1) | unknown, no clinical complaints and not willing to participate in this study |  |
| 5 | 75 | F | Family 2 | cardiomyopathy | none found |
| 6 | unknown | F | Family 2 | no cardiomyopathy, investigated due to other disease |  |
| 7 | unknown | F | Family 2 | no cardiomyopathy, investigated due to other disease |  |
| 8 | 63 | M | Family 3 | cardiomyopathy | missense variants in *TTN* (VUS) |
| 9 | unknown | M | Family 4 | unknown |  |
| 10 | 5 | M | Family 4 | developmental problems, facial dysmorphia, hearing loss |  |
| 11 | 44† | F | Family 5 | ventricular tachycardia, spontaneous coronary artery dissection, inherited connective tissue disease | pathogenic variant found in other gene |
| 12 | 74 | M | Family 6 | cardiomyopathy | none found |
| 13 | 73 | M | Family 7 | cardiomyopathy | none found |
| 14 | 68 | F | Family 8 | cardiomyopathy |  |
| 15 | 31 | F | Family 9 | possibly cardiomyopathy | missense variants in *MYH7* (VUS) and *TTN* (VUS) |

| **Table S2: Primers** |  |  |  |
| --- | --- | --- | --- |
| **Gene** |  | **Forward '5 - '3** | **Reverse '3 - '5** |
| *DSP: splicing in patient cells* | *DSP_ex1-4* | GGACGGCTACTG**TCAAACC** | GACTCGAGGGACACTGATG |
| ,, | *DSP_ex2-5* | **CT**GTCAAACCGGCACGATGTC | TCCAGCTGCCAGCGATAGTC |
| ,, | *DSP_ex2-int2* | AGGCACCAGAACCAGAACAC | CCCAACCCAGGAACAGAAAC |
| ,, | *DSP_ex1-int2A* | GGACGGCTACTG**TCAAACC** | CCCAACCCAGGAACAGAAAC |
| *DSP: in vitro splicing assay* | *DSP_ex2_F_EcoRI:*  *DSP_ex2_R_BamHI* | AAAA**GAATTC**GGGCAT  GGTTATTCTCAAGTGG | AAAA**GGATCC**AGGTGG  CAATCTGTAGTG |
|  | *pSL3 (SD6)* | CTGAGTCACCTGGACAACC |  |
|  | *pSL3 (SA2)* | ATCTCAGTGGTATTTGTGAGC |  |
| *DSP* |  | CAGTGGTGTCAGCGATGATGT | TGACGCTGGATATGGTGGAA |
| *JUP* |  | AGTAGCCACGATGGAGGTGA | AGGTGTATGTCTGCTGCCAC |
| *PKP1* |  | GTACCGGCAAGCTCACATCT | CACCAAGGGATGTCCACACA |
| *PKP2* |  | GCAAATGGTTTGCTCGATTT | GGCTGGTAATCTGCAATGGT |
| *PKP3* |  | GCCATGCGCAACCTCATCTA | AGCTCGAAGATCCCGTTCTC |
| *DSG1* |  | TGAAGGCCCAGTGTTTCGTC | CCTGTGTCCAGGTCAGTAGC |
| *DSG2* |  | TCCACTATGCCACCAACCAC | GCTGGAGCATACACCCTCTC |
| *DSG3* |  | CGTGGTTGTCTCCGCTAGAA | TGGCATCTCACACCGATTGT |
| *DSC1* |  | CCAGTGGTGAAGGCTTAAGGT | TGTTCCAGTGCAAGATCGGC |
| *DSC2* |  | CGGAGATTGTTGCGGTTGA | GGAAAGACGTGCTGCTGTATCA |
| *DSC3* |  | ATGAGGGGCCTGAATGCAC | GTTGATCTTTGACCCCACTGC |
| *KRT1* |  | GACAAGGTGAGGTTCCTGGAG | CAGTTCCGAATCCAACCGAGA |
| *KRT10* |  | GTTCGGGCTCTGGAAGAATCA | GGCATTGTCGATCTGAAGCAG |
| *GAPDH* |  | GCACCGTCAAGGCTGAGAAC | GTGGTGAAGACGCCAGTGGA |

| **Table S3: Antibodies** |  |  |  |
| --- | --- | --- | --- |
| **Protein** | **Clone/ Cat no.** | **Clonality** | **Usage** |
| Desmoplakin I (ROD) | DP2.17 | moAb | WB, IHC-Fr, IF |
| Desmoplakin I&II (N-ter) | DP2.15 | moAb | IHC-Fr, IF |
| Desmoplakin I&II (C-ter) | 2A5 | moAb | IF, IHC-Fr |
| Desmoplakin I&II | ab71690 | poAb | WB |
| Plectin | 10F6 | moAb | WB |
| Plakoglobin | 15F11 | moAb | WB, IHC-Fr, IF |
| Plakophilin-1 | PP1-5C2 | moAb | WB |
| Plakophilin-3 | PKP3-270.6.2 | moAb | WB |
| Desmoglein-1 | ERP6766 | moAb | WB, IF, IHC-Fr |
| Desmoglein-3 | EPR14101 | moAb | WB, IF, IHC-Fr |
| Desmocollin-1 | Dsc1-U100 | moAb | WB, IF, IHC-Fr |
| Desmocollin-3 | Dsc3-U114 | moAb | WB, IF, IHC-Fr |
| Keratin 14 | LL002 | moAb | IHC-Fr, WB |
| Involucrin | 14E1 | moAb | WB, IF, IHC-Fr |
| GAPDH | 10R-G109a | moAb | WB |
| Vinculin | SPM227 | moAb | WB |
